# Allogeneic vs. autologous mesenchymal stem/stromal cells in their medication practice

**DOI:** 10.1186/s13578-021-00698-y

**Published:** 2021-11-02

**Authors:** Chenghai Li, Hua Zhao, Linna Cheng, Bin Wang

**Affiliations:** 1 Stem Cell Program of Clinical Research Center, People’s Hospital of Zhengzhou University, 7 Weiwu Road, Zhengzhou, 450003 China; 2 Institute of Reproductive Medicine, People’s Hospital of Zhengzhou University, 7 Weiwu Road, Zhengzhou, 450003 China; 3 Institute of Hematology, People’s Hospital of Zhengzhou University, 7 Weiwu Road, Zhengzhou, 450003 China; 4 Department of Neurosurgery, People’s Hospital of Zhengzhou University, 7 Weiwu Road, Zhengzhou, 450003 China

**Keywords:** Mesenchymal stem/stromal cell, Single-nucleotide polymorphism, Stem cell heterogeneity, Stem cell microenvironment, Stem cell transplantation

## Abstract

Mesenchymal stem/stromal cell (MSC)-based therapeutics is already available for treatment of a range of diseases or medical conditions. Autologous or allogeneic MSCs obtained from self or donors have their own advantages and disadvantages in their medical practice. Therapeutic benefits of using autologous vs. allogeneic MSCs are inconclusive. Transplanted MSCs within the body interact with their physical microenvironment or niche, physiologically or pathologically, and such cells in a newly established tissue microenvironment may be impacted by the pathological harmful environmental factors to alter their unique biological behaviors. Meanwhile, a temporary microenvironment/niche may be also altered by the resident or niche-surrounding MSCs. Therefore, the functional plasticity and heterogeneity of MSCs caused by different donors and subpopulations of MSCs may result in potential uncertainty in their safe and efficacious medical practice. Acknowledging a connection between MSCs’ biology and their existing microenvironment, donor-controlled clinical practice for the long-term therapeutic benefit is suggested to further consider minimizing MSCs potential harm for MSC-based individual therapies. In this review, we summarize the advantages and disadvantages of autologous vs. allogeneic MSCs in their therapeutic applications. Among other issues, we highlight the importance of better understanding of the various microenvironments that may affect the properties of niche-surrounding MSCs and discuss the clinical applications of MSCs within different contexts for treatment of different diseases including cardiomyopathy, lupus and lupus nephritis, diabetes and diabetic complications, bone and cartilage repair, cancer and tissue fibrosis.

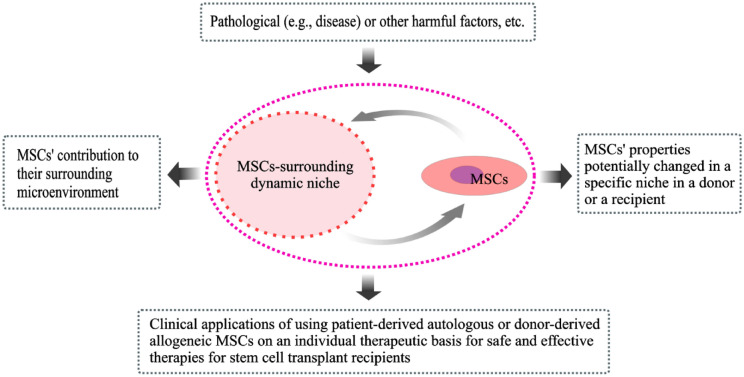

## Introduction

MSCs, referred to as mesenchymal stem/stromal cells, can differentiate towards mesoderm-derived cell lineages such as osteocytes, adipocytes, and chondrocytes [1, 2]. The existence of MSCs in bone marrow (BM) was first suggested by the German pathologist Cohnheim 150 years ago [3]. MSCs were initially described and identified in the 1970s as the discrete “fibroblast” colonies of the BM by Friedenstein et al. [4, 5]. Such cells are currently well known to be localized in the multiple types of adult tissues, including BM, adipose tissue (AT), peripheral blood [2], and human embryo tissues, such as fetal liver [6], fetal BM [7], aorta-gonad-mesonephros and yolk sac [8], as well as various neonatal birth-associated tissues, including placenta, umbilical cord (UC), Wharton’s jelly (WJ) and cord blood [2, 9]. MSCs can originate from perivascular or mural cells as well, i.e., pericytes, from nearly all vascularized tissues [10, 11]. Due to the diverse tissue-specific properties, MSCs derived from different tissues exhibit the varied phenotypic properties and functional behaviors [12, 13]. In the late 1980s, Caplan coined the name “mesenchymal stem cell” based on several key facts such as [14]: (i) embryonic mesenchymal cells in the chick and mouse/human limes; (ii) multi-lineage of mesenchymal cells; (iii) self-renewal and multipotent differentiation in vitro; and (iv) bioactive factors in bone for self-cell repair skeletal defects. Since then, the stem cell properties of “mesenchymal stem cell” remain actively controversial. Given that the multipotency of MSCs in vivo is not known, Caplan proposed to rename the MSCs as Medicinal Signaling Cells in 2010 to more accurately reflect their immunomodulatory and trophic functions [15]. In 2019, the Mesenchymal and Tissue Stem Cell Committee of the International Society for Cellular Therapy (ISCT) suggested a change in nomenclature from “mesenchymal stem cell” to “mesenchymal stromal cell”, which is to further consolidate and clarify ISCT’s MSC committee position on functional definition of mesenchymal stem versus stromal cells [16].

Given their self-renewal and differentiation properties, immunomodulatory capabilities, lacking major histocompatibility complex (MHC) class II molecules, migration and tissue remodeling potential, MSCs have attracted much attention for stem cell-based translational medicine research. The first phase I clinical trial using autologous BM-derived MSCs was conducted by Lazarus et al. in 1995 in 15 patients with complete clinical remission of hematological malignancies [17]. Since then, studies exploring the capability of MSCs in translational medicine are being grown in a remarkable way. Unfortunately, clinical trial failures have frequently appeared for MSC-based therapies [18–20] and, however, rigorously clinical evidence of the therapeutic benefits of MSCs is still lacking. The precise mechanisms of MSCs’ action are not fully understood and there is still a lot to learn.

## Clinical applications of autologous vs. allogeneic MSCs

### Advantages and disadvantages in autologous and allogeneic MSCs

**Fig. 1:**
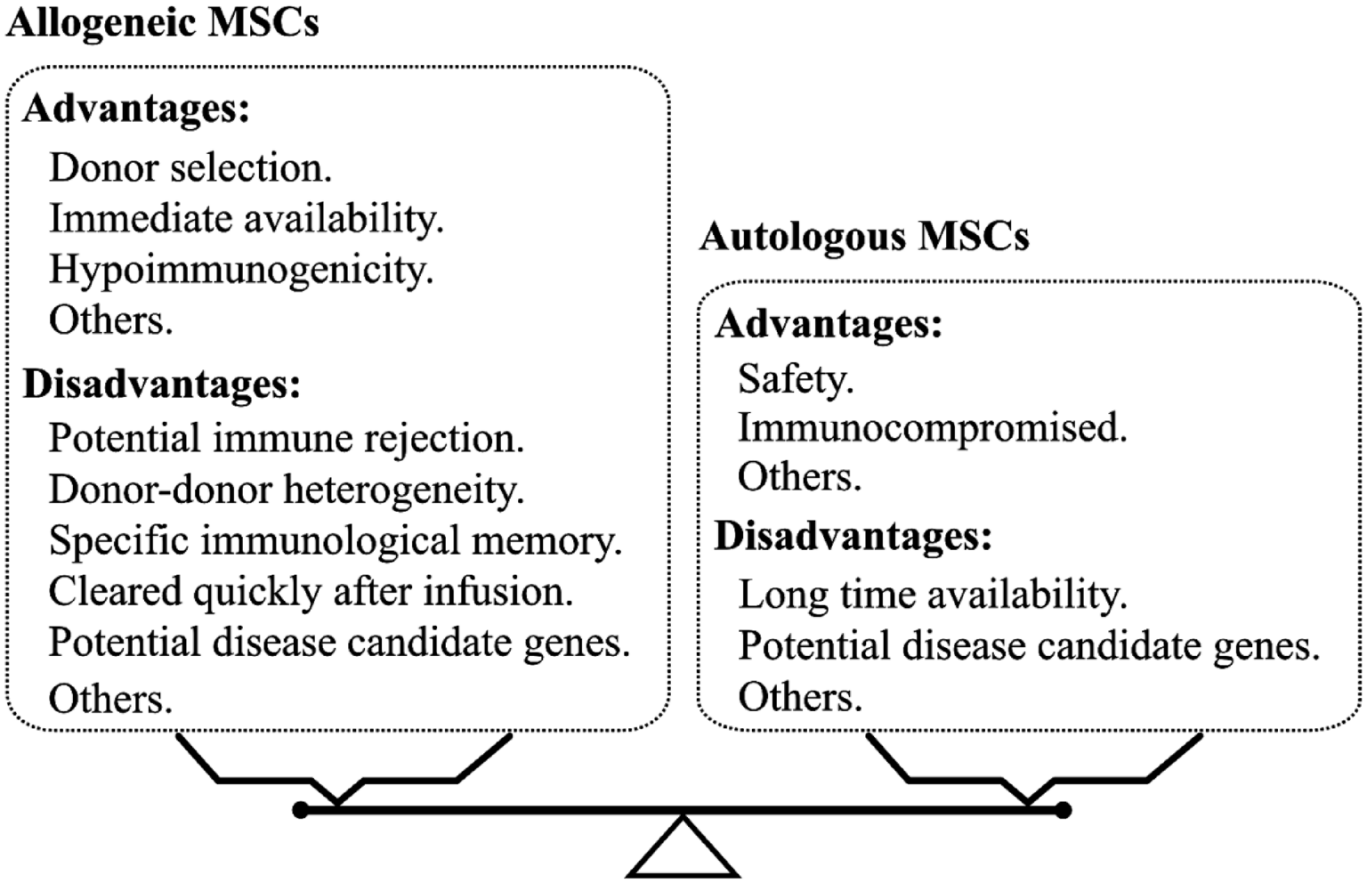
Allogeneic vs. autologous MSCs: advantages and disadvantages. MSCs obtained from donors and self have their own advantages and disadvantages

Clinical applications of autologous and allogeneic MSCs are already available for treating a range of diseases or conditions. Autologous MSCs are easy to obtain and lacking of immune rejection after infusion. Nevertheless, autologous MSCs require a few weeks for isolation, in-vitro expansion and release and patient-derived autologous MSCs may underlie systemic diseases. Allogeneic MSCs can offer several advantages such as donor selection, various sources, low immunogenicity, and off-the-shelf availability. Allogeneic MSCs may be also immunogenic and such cells can induce an immune memory response under appropriate condition [21–23], albeit MSCs have been believed to be immune-privileged or immunocompromised. Joswig et al. conducted an in vivo study to assess the clinical response to repeated intra-articular injection of autologous and allogeneic MSCs and found a significant adverse response of the joint to allogeneic MSCs after a second injection, suggesting an adaptive immune response to the injected allogeneic MSCs but not autologous MSCs [24]. In contrast, Huang et al. [25] observed that the implanted allogeneic MSCs expressed the high levels of MHC-Ia and MHC-II by 14 days in an myocardial infarction (MI) rat model after cell implantation and therapeutic benefits were lost within 5 months, which also suggests a transition from an immunoprivileged to an immunogenic state after differentiation of MSCs. Currently, allogeneic MSC therapy is increasing in clinical translational field and these cells have been shown to be clinically safe and effective. To minimize any potential anti-donor immune responses, several strategies are suggested by Lohan et al. in their systematic review [26], including the use of immunosuppressive drugs. However, the potential risks and limitations of using autologous vs. allogeneic MSCs for therapeutic applications are still highly debated such as the potential impact of donor–donor heterogeneity. In general, allogeneic and autologous MSCs have their own advantages and disadvantages in the preclinical and clinical practice (Fig. 1).

### Short-term lifespan and benefit of infused MSCs

Owe to MHC-unrestricted property of MSCs, a number of clinical trials using allogeneic MSCs or MSC-based therapeutic products are being carried out for treatment of a variety of medical conditions. Given the low engraftment efficiency of MSCs, only a limited number of such cells can migrate and reach the disease target sites after systemic transplantation [27–29]; thus limit their clinical efficacy. Pulmonary passage seems to be a major obstacle for intravenous MSCs delivery for regenerative tissue therapy in preclinical studies [30, 31]. Those in vivo studies suggest that MSCs exert their therapeutic influence through the secretion of soluble protein/peptide molecules. MSCs have a short-term lifespan after systemic infusion and the most circulating MSCs, allogeneic or even autologous, will be lysed by the humoral components and immune cell subsets [32]. While a large number of in vivo studies have shown the short lifespan of MSCs through tracking intravenously administered MSCs, clinical data are rare for tracking MSC homing into different tissues within the transplanted patients. von Bahr et al. previously examined autopsy material from 18 patients who were infused with MSCs and 108 tissue samples from 15 patients were analyzed for MSC donor DNA to evaluate engraftment of MSCs [33]. MSC donor DNA was detected in 9/13 MSC infusions within 50 days from MSC infusion to sample collection and in 2/8 earlier MSC infusions within 75 and 87 days, respectively. A negative correlation was observed between the detection of MSC donor DNA and the time from MSC infusion to sample collection [33]. Consequently, the findings in this study indicate that systemically administered MSCs have a relatively short life in the recipients, suggesting that MSCs may exert their short-term therapeutic benefits.

In specific contexts, therapy with MSCs can improve short-term recovery for diseases or conditions such as acute respiratory distress syndrome (ARDS). ARDS is associated with acute inflammatory lung injury, lung permeability and edema [34] and hospital mortality in patients with ARDS remains high with 34.9% for those with mild, 40.3% with moderate, and 46.1% with severe ARDS [35]. Hospitalized severe patients with coronavirus disease 2019 (COVID-19) pneumonia require to be treated in the intensive care unit (ICU) due to pneumonia complications, including 61.1% of these patients with ARDS [36]. There is a growing interest in using of MSCs or MSC-derived therapeutic products as a potential new treatment for ARDS. However, the precise mechanisms of action of MSCs remain to be fully investigated. A recent systematic review highlights several potential therapeutic mechanisms of MSCs in ARDS [37], including immunomodulatory effects on immune and inflammatory cells, maintaining the alveolar epithelial and endothelial barrier through paracrine factors secreted by MSCs, reducing endoplasmic reticulum stress, and anti-fibrotic potential of MSCs in ARDS. Two recent phase 1/2a randomized controlled clinical trials report the therapeutic benefits of using UC-derived MSCs in subjects with COVID-19 ARDS mainly through anti-inflammatory and immunomodulatory activities [38, 39], which indicates a set of inflammatory cytokines downregulated at the day 6 after infusion. The therapeutic potential of MSCs has been observed in a case series study, which suggests the improved PaO_2_/FiO_2_ ratio, the ratio of arterial oxygen partial pressure to fractional inspired oxygen, in severe COVID-19-induced ARDS patients in ICU with critically hypoxemia [40]. Due to the physical properties of MSCs, the issue of exogenous MSC engraftment after infusion remains actively controversial. To avoid cell-related problems, MSC-derived exosomes have attracted great interest in recent years in translational biomedicine field. One open-label cohort study conducted by Sengupta et al. [41] demonstrates the clinical presentation and oxygenation improved in severe COVID-19 patients with moderate-to-severe ARDS after treatment with exosomes secreted by BM-derived MSCs. To extend our discussion, MSCs cultured under hypoxic condition have a high expression of chemokine stromal-derived factor-1 receptors, CXCR4 and CXCR7, to promote MSCs’ migration [42]. When MSCs are cultured under long-term (10 days) hypoxia, such cells downregulate their surface markers including CD44 and CD105 [43]. Theoretically, exogenous MSCs may exert their short-term effects to improve ARDS or other infectious diseases through the immediate anti-inflammation and immunomodulation and this is also specific therapeutic characteristic of MSCs. However, therapy with MSCs for ARDS still confronts many challenges including safety issues, low survival ability, engraftment and migration after infusion as well as the optimized cell preparation, dose, infusion route, study subjects, and the window period.

### Therapeutic effects of autologous vs. allogeneic MSCs

Autologous and allogeneic MSCs have their own advantages and disadvantages and, on an individual therapeutic basis, clinical applications of autologous or allogeneic MSCs need to be designed to maximize their therapeutic activity while to minimize their potential side effects. In this section, we summarize the clinical applications using autologous vs. allogeneic MSCs in various fields of translational biomedicine (Table 1). We then extend our discussion and analyze a bidirectional interaction between the transplanted autologous or allogeneic MSCs and their existing harmful or non-harmful niche environments (as will be discussed later). Finally, we conclude with a summary of therapeutic limitation of using autologous or allogeneic MSCs for long-term beneficial therapies for the stem cell transplant recipients.

**Table 1:** Summary of clinical studies with autologous and allogeneic MSCs

Disease/condition	Auto/allo	Source	Study category/	Single dose of MSCs	Therapeutic effect	References
			Phase	(× 10^6^ cells)		
Cardiomyopathy	Auto	BM	Cohort trial	0.5–1.0/kg or 2.0–3.0/kg	Improvement of clinical symptoms and left ventricular function in patients with chronic severe refractory dilated cardiomyopathy	[44]
	Auto	BM	Randomized and controlled trial	3.08 ± 0.52	No improvement in myocardial viability and function in acute STEMI patients	[45]
	Auto	BM	Phase I trial	N/A	Cardiac function and quality of life improved in patients with ischemic heart disease undergoing cardiac surgical revascularization at one-year follow-up	[46]
	Auto	BM	Phase I/II trial	61.5/10–16 viable sites	Improvement in cardiac performance, left ventricular remodeling, and patient quality of life	[47]
	Auto	BM	Randomized and controlled trial	77.5 ± 67.9 (inter-quartile range 53.8)	Improvement in end-systolic volume, EF, stroke volume, cardiac output and myocardial mass at 6 months follow-up in MSC-treated patients with chronic ischaemic heart failure	[48]
	Allo	BM	Phase I trial	Dose-ranging (0.5, 1.6 and 5/kg)	Improvement in left ventricular EF and remodeling in MSC-treated patients with acute myocardial infarction	[49]
	Allo vs. auto	BM	Phase I/II	100/10 LV sites	Safety of the intervention of allo or auto MSCs and great improvement in EF, 6MWT, MLHFQ, and endothelial restoration in allo compared to auto MSC injection in patients with chronic non-ischemic dilated cardiomyopathy	[50]
	Allo vs. auto	BM	Phase I/II	Serially escalated: 20, 100, or 200/10 LV sites	Improvement in functional status and quality of life in patients with ischemic cardiomyopathy	[51]
Lupus/lupus nephritis	Auto	BM	Case series study	N/A	No change in SLE activity indexes but increase in T regulatory cells during 14 weeks of follow-up	[57]
	Allo	BM	Case series study	1.5/kg	Complete or partial remission of SLE after MSC infusion through a 9-month follow-up	[61]
	Allo	BM	Phase I/II	1.0/kg	Improvement of clinical outcomes and decrease of serological autoimmune markers at 1-year follow-up	[62]
	Allo	UC	Phase I/II	1.0/kg	Mixed clinical outcomes presented, as showed 32.5% and 27.5% of patients with MCR and PCR to MSC infusion, respectively, and 12.5% and 16.7% of patients with disease relapse at 9 and 12 months of follow-up, respectively	[63]
DM/DM complication	Auto	BM	Randomized and controlled trial	N/A	Improvement of healing in type 2 DM patients with critical limb ischemia after 24 weeks of follow-up	[70]
	Auto	BM	Phase I	N/A	Safe and effective therapeutic option for Bullosis diabeticorum	[71]
	Auto	BM	Pilot trial	3/kg	Therapeutic safety and effectiveness in diabetic retinopathy	[72]
	Allo	BM	Phase I/II	Dose-escalating, 0.3, 1.0 or 2.0/kg	Safety and feasibility of MSC therapy for type 2 DM during a 12-week period	[73]
	Allo	WJ	Phase I/II	1.0/kg	Therapeutic potential in type 2 DM, as showed the improvement in laboratory parameters and systemic inflammation	[74]
	Allo	BM	Phase I/II	150 or 300	Improvement in glomerular filtration rate at 12 weeks post-infusion in patients with DM nephropathy	[75]
	Allo	UC	Phase I/II	1.1/kg	Metabolic improvement in type 1 DM patients treated with MSC transplantation in combination with auto BM mononuclear cells	[76]
	Allo vs. auto	AT	Open-labeled and two-armed trial	103.14 mL with 2.65 ± 0.8 × 10^4^ ISCs/kg or 95.33 mL with 2.07 ± 0.67 × 10^4^ ISCs/kg	Reduction in insulin requirement and a better long-term hyperglycemia control in type 1 DM patients treated with co-infusion of auto insulin-secreting MSCs and BM-derived HSCs, compared with allo stem cell therapy	[77]
Bone/cartilage repair	Auto	BM	Phase I/II	10 or 100	Clinical and functional improvement of knee osteoarthritis after intra-articular injection of MSCs versus hyaluronic acid during follow-up of 12 months	[83]
	Auto	BM	Phase I/II	10 or 100	Clinical and functional improvement of knee osteoarthritis after intra-articular injection of MSCs versus hyaluronic acid during follow-up of 4 years	[84]
	Auto	AT	Phase IIb	100	Clinical and functional improvement and pain relief in patients with knee osteoarthritis at 6 months of follow-up	[85]
	Auto	AT	Phase IIb	50	Clinical and functional improvement and cartilage regeneration in patient with knee osteoarthritis at 12 months of follow-up	[86]
	Allo	BM	Phase II	Dose escalation: 20, 50, 75, or 150	Treatment of knee osteoarthritis with a twenty-five-million-cell dose of MSCs shown a trend toward pain reduction	[87]
	Allo	BM	Phase I/II	40	Improvement of both pain and cartilage quality without major adverse events in patients with knee osteoarthritis	[88]
	Allo	AT	Randomized and controlled trial	3.9 or 6.7	Improvement in pain scores and quantitative MRI assessments	[89]
	Allo	Placenta	Pilot trial	50–60	Safety in intra-articular injection of MSCs and clinical improvements at 24-week follow-up	[90]
	Allo	UC	Phase I/II	20	Improvement in pain and clinical outcomes in osteoarthritis patients	[91]
Cancer	Auto	BM	Phase I/II	1.0–2.2/kg	Rapid hematopoietic recovery after co-infusion of auto MSCs and auto peripheral blood progenitor cells in patients with advanced breast cancer	[94]
	Auto	BM	Phase I	1.0 or 1.5/kg	Safety and feasibility of MSC infusion in combination with ganciclovir in patients with advanced gastrointestinal adenocarcinoma	[95]
	Auto	BM	Phase I/II	3.0/kg	Safe and tolerable treatment with MSC infusion in combination with ganciclovir in patients with advanced gastrointestinal adenocarcinoma	[96]
	Allo	BM	Phase I	1.0 or 2.0/kg	Safe MSC infusion in prostate cancer patients but no homing of MSCs to the primary tumors at sufficient levels	[97]
	Allo	UC	Cohort study	1.14/kg	Enhancement of hematopoietic recovery and reduction of GVHD incidence in acute leukemia children when co-transfusion of MSCs and HSCs	[98]
	Allo	UC	Cohort study	N/A	Enhancement of hematopoietic recovery when co-transplantation of MSCs and cord blood in high-risk leukemia patients	[110]
	Allo	BM	Retrospective study	6.81/kg	Response rate to MSC infusion among 50% patients with steroid-refractory acute GVHD III/IV	[111]
Tissue fibrosis	Auto	BM	Phase II	50	Histological improvement in 54.5% patients with alcoholic liver cirrhosis following MSC therapy	[112]
	Auto	BM	Phase I	100	Improvement in laboratory parameters such as liver function and quality of life for patients with liver cirrhosis	[113]
	Auto	BM	Phase II	50	Reduction of hepatic fibrosis and improvement of liver function in patients with liver cirrhosis	[114]
	Allo	BM	Phase I	20, 100, or 200	Safety of a single of MSC infusion up to 2 × 10^8^ cells/infusion in IPF patients	[115]
	Allo	Placenta	Phase Ib	1.0 or 2.0/kg	Feasibility and short-term safety of MSC infusion in patient with IPF	[116]
	Allo	UC	Phase I/II	1.0/kg	Improvement of lung function and computed tomography imaging after MSC infusion combined with plasmapheresis in systemic sclerosis patients	[117]

*6MWT* Six Minute Walk Test, *Allo* allogeneic, *AT* adipose tissue, *Auto* autologous, *BM* bone marrow, *BMDM* diabetes mellitus, *EF* ejection fraction, *HSCs* hematopoietic stem cells, *IPF* idiopathic pulmonary fibrosis, *ISCs* insulin-secreting MSCs, *kg* kilogram body weight, *LV* left ventricular, *MCR* major clinical response, *MLHFQ* Minnesota Living with Heart Failure Questionnaire, *MRI* magnetic resonance imaging, *N/A* not available, *PCR* partial clinical response, *SLE* systemic lupus erythematosus, *STEMI* ST-segment elevation myocardial infarction, *UC* umbilical cord, *WJ* Wharton’s jelly

#### Cardiomyopathy

Clinical trials have shown that the therapeutic benefits of using autologous vs. allogeneic MSCs are inconclusive, while therapy with such cells appears to be undoubtedly safe. One early clinical study reported that intramyocardial or intracoronary autologous BM-derived MSC treatment was safe and effective for chronic severe dilated cardiomyopathy (DCM) [44], as showed the improvement of left ventricular function and scar reduction in these patients. However, this trial for autologous MSCs was limited by the small sample size and also lacked a control arm. Gao et al. [45] previously designed a randomized and multicenter trial to assess 2-year follow-up safety and efficacy of autologous BM-derived MSCs for treatment of acute MI. This study by Gao et al. showed that, compared with baseline, improvement of myocardial ischemia in patients treated with intracoronary infusion of autologous BM-derived MSC as well as in the control group with standard medical treatment. Of note, no significant difference was observed between the both groups about myocardial viability and function in the clinical setting [45]. Preliminary positive results in other studies suggested that autologous BM-derived MSCs are safely and effectively administered to treat patients suffering from ischemic heart diseases [46–48].

Therapeutic safety and efficacy of using allogeneic BM-derived MSCs was reported in a randomized, double blind, placebo-controlled clinical trial for treatment of acute MI [49], as showed the improvement in left ventricular ejection fraction and remodeling in MSC-treated patients. In contrast, one previous POSEIDON randomized trial was designed to test the safety and efficacy of allogeneic vs. autologous MSCs in patients with non-ischemic DCM [50]. Based on clinical results in the study [50], allogeneic MSCs were seemly to be superior to the self-derived MSCs, as illustrated significant improvement in ejection fraction, Six Minute Walk Test, Minnesota Living with Heart Failure Questionnaire scores, and endothelial function. In another POSEIDON randomized trial, allogeneic and autologous BM-derived MSCs were delivered via transendocardial injection in 30 patients with ischemic cardiomyopathy [51]. The study demonstrated that therapy with allogeneic and autologous MSCs improved functional status and quality of life in these patients [51] and no difference was observed between the cell types.

As aforementioned, therapy with MSCs, autologous or allogeneic, improves left ventricular ejection fraction, decreases scar size, reverses ventricular remodeling along with eliciting the cell secretion of paracrine factors, although the exact mechanism of action of MSCs remains to be further investigated. However, therapeutic benefit is modest and there are frequently combined clinical results for MSC intervention in patients with ischemic cardiomyopathy [52, 53]. Of note, the microenvironment in infarction heart may be harmful to transplanted MSC survival due to high concentration of free radicals [54, 55]. One previous in vivo study showed that intro-myocardial injection of bone marrow cells (BMCs) from post-MI donor mice led to impaired therapeutic efficacy of BMCs for treatment of MI [56], which indicates impairment of BMCs by severe donor MI. The study further deliberated that MI induced inflammatory state and pro-inflammatory alteration of bone marrow composition [56]. Of clinical relevance, this study suggests that implantation of autologous BMCs, in contrast, is likely to be less efficacious.

#### Lupus and lupus nephritis

Transplanted autologous BM-derived MSCs were not shown to be clinically efficacious in response to treatment through the week 14 in two system lupus erythematosus (SLE) patients, albeit no adverse effects were noted [57]. An early in vitro and in vivo study showed that BM-derived MSCs from SLE patients had the abnormalities of cytokine expression profiles and the population doubling time [58]. BM-derived MSCs from SLE patients also demonstrated the early sign of senescence, the increased telomerase activity [59]. Gene expression profile in another study also revealed the biological abnormalities of BM-derived MSCs from SLE patients, such as actin cytoskeleton, cell cycling regulation, bone morphogenetic protein-5 as well as activated mitogen-activated protein kinase and dysregulation in transforming growth factor-β signaling pathways [60].

Therapeutic potential of allogeneic BM-derived MSCs for lupus nephritis was reported in an SLE case carries study [61]. In this clinical trial [61], three SLE patients with class IV active proliferative nephritis were treated with allogeneic BM-derived MSCs and SLEDAI (SLE disease activity index) scores revealed that disease remission were complete for two patients and partial for the third one after 9 months of follow-up. Therapy with allogeneic BM-derived MSCs was also reported in a pilot clinical trial [62]. This study demonstrated the clinical improvement in 12 of 13 patients with a marked decrease in the SLEDAI score at 12-month follow-up and the decreased serum titres of anti-dsDNA antibody, one of SLE marker auto-antibodies, from baseline for 1 month and 3 months post transplantation, respectively [62]. A multicenter clinical study showed that transplanted allogeneic UC-derived MSCs were safe and effective in severe and refractory SLE and, however, therapeutic effect may be not permanent, reflected that 12.5% and 16.7% of SLE patients had disease relapses after 9 and 12 months of follow-up, respectively [63].

SLE is an autoimmune disease characterized by the multiple of autoantibody production and the multiple of organ complications. Therapy with MSCs from healthy donor individuals without relation to genetic variants is increasing in prevalence in SLE. Prior studies have shown the genetic factors contributing to MSC dysfunction in SLE [64–66]. For instance, HLA-DM and HLA-G are identified in SLE [67] and HLA-G is associated with immunosuppressive property of MSCs [68]. Patient self-derived MSCs are believed to have impaired immunosuppressive capacity in innate and adaptive immune responses partly due to the abnormal genetic background [69]. In this regard, autologous MSCs may not be eligible for therapeutic option in SLE.

#### Diabetes mellitus (DM) and DM complication

MSCs have also shown therapeutic potential for DM and DM complications. Both autologous and allogeneic MSCs are widely used for treating individuals with type 1 and 2 DM (T1DM and T2DM). Therapeutic potential of autologous BM-derived MSCs revealed in T2DM critical limb ischemia and foot ulcer [70] and lower limb bullosis diabeticorum [71]. The use of autologous BM-derived MSCs for the treatment of diabetic retinopathy was also evaluated in a pilot clinical trial and this study suggested autologous MSCs as a potentially safe and effective treatment option for diabetic retinopathy [72]. Laboratory parameters and clinical trial data in the trial [72] showed a significant decrease in the levels of fasting blood glucose and serum C-reactive protein from baseline at 1-, 3-, and 6-month follow-up and a significant improvement in best corrected visual acuity after 3 and 6 months, respectively.

Clinical data from T2DM individuals documented safety and effectiveness of allogeneic BM-derived MSCs [73] and WJ-derived MSC [74]. Allogeneic BM-derived MSCs were also shown to be safe and improved diabetic nephropathy complication after administration in a randomized and placebo-controlled clinical study [75]. A previous pilot randomized controlled clinical study was conducted by using allogeneic UC-derived MSCs combined with autologous BM cells, a cell-based combination therapeutic approach, to determine the safety and effectiveness in established T1DM [76]. This study suggested that co-transplantation of the allogeneic UC-derived MSCs and autologous BM cells was safe and may lead to moderate metabolic improvement in T1DM patients. There was another open-labeled and two-armed trial for T1DM using allogeneic and autologous AT-derived insulin-secreting MSCs together with BM-derived hematopoietic stem cells (HSCs) [77]. Co-transplantation of autologous MSCs and HSCs showed a better response in T1DM individuals as compared with the allogeneic group [77]. Noteworthily, the synergistic combination approaches using stem cells from autologous and allogeneic sources need to validate from the large studies.

Persistent hyperglycemic milieu can alter MSCs’ properties and may further affect their therapeutic potential in DM patients. AT-derived MSCs from diabetic donors, compared to MSCs from non-diabetic individuals, showed higher levels of cellular senescence and apoptosis as well as the reduction of osteogenic and chondrogenic differentiation potential [78]. Autologous AT-derived MSCs from T2DM individuals exhibited the reduced proliferation and inhibited migration and homing to sites of inflammation in a previous clinical report [79] and those cells displayed the reduced fibrinolytic activity [80], thus, increasing the probability of developing thrombosis for T2DM patients. Additionally, T1DM donor MSCs exhibited to be phenotypically and functionally similar to the health donor MSCs [81]. Furthermore, MSCs derived from T1DM patients can maintain their normal capability of secretion and immunomodulation and, however, MSCs from T2DM individuals may be usually dysfunctional such as the increased rates of senescence and apoptosis and the decreased proliferation and angiogenesis potential [82]. Therefore, while the current studies indicate that using autologous MSCs is likely to be suitable for T1DM therapy, the large scale trials still need to test autologous vs. allogeneic MSCs’ safety and efficacy in T1DM as well as T2DM.

#### Bone and cartilage repair

MSCs, a non-HSC population, were first identified by Friedenstein and colleagues in BM [4, 5] and MSCs are being routinely explored in clinical trials for treatment of bone and cartilage diseases due to MSCs’ immunomodulatory properties and multipotential differentiation. Autologous MSCs have achieved a promising therapeutic effect on the treatment of osteoarthritis (OA) disease. Lamo-Espinosa et al. [83] conducted a phase I/II multicenter randomized clinical trial with one active control (hyaluronic acid alone) through intra-articular injection of autologous BM-derived MSCs for OA patients and their study showed a clinical and functional improvement of knee OA during 12 months of follow-up. The clinical trial continued to be observed by the same team in patient with OA who had been treated with autologous BM-derived MSCs as a safe and effective therapeutic option after a follow-up of 4 years [84]. Still, two controlled and randomized phase IIb clinical trials demonstrated that the treatment of knee OA with intra-articular injection of autologous AT-derived MSCs resulted in improvement in joint function and pain relief for those patients [85, 86].

Consistent with intra-articular injection of the autologous MSCs, the local intra-articular injection of allogeneic MSCs has been reported to be safe and effective for treatment of chronic knee OA in previous clinical studies. A phase II clinical study was conducted to assess the safety and efficacy of intra-articular injection of allogeneic BM-derived MSCs to OA patients who received different doses of cells at 25, 50, 75, and 150 million, respectively [87]. Short-term local pain and swelling, the most common adverse events, were observed in the higher dose groups (75 or 150 million group) and these adverse events recovered with the symptomatic treatment [87]. Compared to the other groups of OA patients, the 25-million-cell dose group presented a trend towards pain reduction, thus suggesting an optimized dose of MSCs. Another randomized controlled multicenter phase I–II trial was also conducted for treatment of knee OA with allogeneic BM-derived MSCs [88]. This study demonstrated that allogeneic BM-derived MSC transplantation is safe and effective for cartilage repair, as evidenced by the quantitative magnetic resonance imaging that indicates the healing of partial articular cartilage and no major adverse events. The safe and effective therapies were also reported in the clinical repair of knee OA using allogeneic AT-derived MSCs [89], placental MSCs [90] and UC-derived MSCs [91].

MSCs have been the subject of stem cell-based clinical trials for the treatment of different types of skeletal diseases or medical conditions over the last few decades. However, the clinical use of the OA patient-derived MSCs to repair bone and cartilage defects remains a major challenge. An in vitro study showed that OA condition and older age may affect the multipotential of the retropatellar fat pad-derived MSCs (RFMSCs) from OA patients [92]. This clinical study by Chua et al. [92] demonstrated, in contrast, the lower tri-lineage differentiation potential of RFMSCs as well as a significant decrease in the expression of stemness genes such as *Sox2*, *Rex1*, *Nanog3*, *Oct4* and *Nestin*. Another in vitro study by Murphy et al. showed that the chondrogenic and adipogenic activity was reduced in BM-derived MSCs from OA patients [93]. Interestingly, there was no decline in vitro with osteogenic activity of BM-derived MSCs obtained from OA patients [93], thus suggesting that BM-specific MSCs better serve their purposes of the homeostatic maintenance of MSCs-derived BM. However, MSCs from OA donors appear to be deficient and underlying mechanisms of such cell-mediated bone healing in diseased microenvironments still remain to be fully demystified.

#### Cancer

As noted above, the first clinical trial reported the safety of infusion of autologous BM-derived MSCs for treatment of hematological malignancies [17]. The same team conducted another phase I/II trial of the infusion of autologous BM-derived MSCs for breast cancer patients at the time of transplantation of autologous peripheral blood progenitor cells (PBPCs) [94]. The authors of this trial [94] reported that co-infusion of autologous MSCs and PBPCs was safe and led to rapid hematopoietic recovery in breast cancer patients. Another group conducted a phase I clinical study using genetically modified autologous MSCs in combination with Ganciclovir (GCV) to evaluate the safety and efficacy for the treatment of advanced gastrointestinal adenocarcinoma (AGIA) [95]. The application of autologous MSCs was performed to express HSV-TK that phosphorylates GCV generating a toxic metabolite [95, 96] to suppress cancer growth. The MSC-based combination approach for cancer therapy demonstrated the safety and tolerability in patients with AGIA and, in terms of effectiveness, the stable disease in 4/6 patients and the progressive disease in 2/6 patients presented after treatment [95]. The same group conducted a subsequent open-label multicenter phase I/II trial using the same therapeutic strategy for AGIA treatment and reported that 5/10 patients achieved stable disease and, however, the levels of any tumor markers did not change after the treatment [96].

A phase I clinical trial was performed to test the safety and cancer-homing ability of allogeneic BM-derived MSCs for 4–6 days following the MSC systemic infusion prior to prostatectomy [97]. The clinical trial by Schweizer et al. [97] demonstrated that systemically infused allogeneic BM-derived MSCs were safe in patients underwent prostate cancer. However, in their study, MSCs were undetectable in all subjects via the analysis of donor and recipient profiles of single-nucleotide polymorphisms (SNPs), a measurement of the relative amount of donor DNA versus recipient DNA in the prostate specimen, and, as such, this study was stopped early. Another clinical study demonstrated that co-transfusion of UC-derived MSCs and allogeneic HSCs led to effective hematopoiesis, as showed improvement of neutrophil and platelet recovery in Children with high-risk acute leukemia and, importantly, allogeneic UC-derived MSCs could reduce the incidence and severity of severe graft-versus-host disease (GVHD) [98].

Tumors, as non-homogeneous masses, are very heterologous. As known, tumor has been described as a type of unhealed wound highly associated to inflammation [99]. Prior studies have shown that MSCs are presented in the tumor microenvironment (TME) or in primary tumors, such as hepatocellular carcinoma [100], breast cancer [101], osteosarcoma [102], and prostate cancer [103]. In addition, MSCs are an integral cellular component of the dynamic TME and such cells are able to migrate and reside in the tumor-associated stroma in response to multiple signals [104–106]. MSCs play a dual role of promoting tumor growth and inhibiting tumor development [9]. For example, MSCs can contribute to establishment of a pro-tumorigenic environment for tumor cell homing and proliferation in the bone marrow [107]. Thus, clinical studies should be designed in the anti-tumor setting to consider that MSCs could actually exert their tumor-promoting effects. Given MSCs’ tropism to the site of inflammation or wound healing, it is likely that MSCs play a role in tissue maintenance and regeneration. As such, different therapeutic approaches for targeting delivery of various anti-cancer biologic agents/compounds have been shown in animal models for solid tumor treatment [9, 107].

However, MSCs display the distinct anti-tumor properties in liquid tumors/leukemia vs. solid tumors. MSCs are able to promote hematopoietic recovery by enhancing engraftment and reduce GVHD incidence through their immunomodulatory and anti-inflammatory properties [108, 109]. In a previous clinical study, the hematopoiesis was shown to be faster in patients with high-risk leukemia received co-transplantation of UC-derived MSCs and cord blood compared to the cord blood transplantation alone [110]. Another previous clinical study reported that MSC infusion led to an overall response rate of 50% of the patients with acute GVHD III/IV after HSC transplantation refractory to corticosteroids [111]. In general, clinical data available are still lacking regarding the functional properties of MSCs, autologous or allogeneic, in the anti-tumor clinical research.

#### Tissue fibrosis

Clinical studies have proposed that using autologous or allogeneic MSCs as a novel anti-fibrotic cytotherapy approach in different tissue types of fibrosis. A previous phase II clinical trial was conducted to assess anti-fibrotic effect of autologous BM-derived MSCs in a small size of cohort of 11 patients with alcoholic cirrhosis [112], an advanced stage of progressive hepatic fibrosis. The Laennec fibrosis scoring analysis following transplantation revealed that histological improvements were observed in 54.5% patients given 5 × 10^7^ MSCs with 2 separate infusions and 4 weeks apart [112]. Similarly, clinical trials also showed that autologous BM-derived MSC administration reduced histological fibrosis and improved liver function in those patients with alcoholic cirrhosis [113, 114]. Consequently, BM-derived MSC treatment can be a suitable option for fibrosis reduction and recovery from liver injury in patients with alcoholic cirrhosis. Clinical data available suggest that MSC-based anti-fibrotic therapies are likely to be safe without adverse effects after MSC transplantation and may improve recovery of disease, but efficacy is modest in the treatment and prevention of tissue fibrosis.

A previous phase I clinical trial reported the safety and effectiveness of allogeneic BM-derived MSCs in patients with mild-moderate idiopathic pulmonary fibrosis (IPF) [115]. Glassberg et al. [115] conducted a single intravenous infusion at a dose of 20, 100, or 200 × 10^6^ MSCs, respectively, and found that allogeneic BM-derived MSCs were well tolerated in IPF up to 2 × 10^8^ cells/infusion and no serious side effects or complications were identified. Another phase Ib clinical study was conducted by using of placenta-derived MSCs and showed the feasibility and short-term safety of MSC infusion in patients with IPF [116]. Clinical data available support the safety of the allogeneic MSCs as a potential therapy for IPF and remain challenging for future efficacy studies. A previous clinical study also suggested that treatment with allogeneic UC-derived MSCs in combination with plasmapheresis may benefit patients with systemic sclerosis (SSc) [117], an autoimmune connective tissue disease characterized by chronic inflammation and fibrosis of the skin and internal organs, including pulmonary fibrosis [118]. Another clinical trial effort using allogeneic BM-derived MSCs is currently underway in patient with digital ulcers in SSc [119].

Fibrosis is the formation of excessive fibrous tissues or scars in the context of the both physiological and pathological wound healing and tissue modeling. Tissue damage and inflammation are common characteristics of tissue fibrosis. Inflammation-mediated fibrosis development in many types of fibrotic diseases by a variety of activated inflammatory cells/molecules is summarized in a systematic review [120]. To this end, MSCs may thereby exert anti-inflammation and immunomodulatory effects on fibrosis development. On the other hand, MSCs exist in the perivascular niche microenvironment [10] and the perivascular MSC-like cells are involved in tissue fibrosis, such as renal and heart fibrosis [121]. MSCs are identified in the keloid scar tissue and such MSCs can maintain their differentiation potential into adipocytes and osteocytes [122]. MSCs can also be identified in adult human lung tissues obtained from patients with IPF [123]. Importantly, it has been well documented that fibrosis tissue-resident mesenchymal cells, including fibroblasts, myofibroblasts, smooth muscle cells, and MSCs, potentially contribute to the fibrosis development [124, 125]. MSCs have the potential of transformation to the above mesenchymal cells (fibroblasts, myofibroblasts, smooth muscle cells) [122, 124, 126, 127] to play a suspected dual role in physiological and pathological tissue fibrotic processes. Therefore, due to the plasticity of MSCs, such cells may play a dual role in fibrosis development and improvement. The benefits of MSCs as a potential therapeutic option for fibrosis disease need to be carefully balanced with their potential risks in clinical settings.

## MSC-associated dynamic niches

Clinical and preclinical studies to test the safety and effectiveness of MSCs or MSC-derived therapeutic products need to put them into a foreign tissue microenvironment in the recipients. Therefore, MSCs within the transplanted tissue interact with their special local microenvironment or niche, physiologically or pathologically, to maintain their unique biological properties. Biological behaviors of MSCs may also be affected by a newly established niche microenvironment and vice versa. A temporary microenvironmental state may also be altered to establish a new niche microenvironment. There are discrete sub-niches in different types of tissues within the body, where such MSCs are physically existing in or MSC-surrounding, including physiological, pathological, or physio-“mixed” with pathological (pathophysiological or physiopathological) microenvironments. Indeed, the issue of separating different MSCs’ microenvironments is challenging since the diverse MSC-associated tissue niches are dynamic and temporal. Mostly, such MSCs are existing in or surrounding the very heterogeneous microenvironments (e.g., TME), which is likely to imply harmful and/or non-harmful factors in the niches that effect on MSCs’ biology at the same time. An example of the MSC-associated dynamic microenvironment, BM niche, is used in the present paper to further illustrate this point.

BM stroma is comprised of multipotent progenitors including HSCs and MSCs [128, 129]. Usually, stem cells reside in their newly established niche microenvironment once a stem cell niche is formed. In theory, the infused or endogenous MSCs can home and migrate into the sites of tissue injury in response to multiple signals. MSCs were first proposed to reside in bone marrow [4, 5]. Physiologically, MSCs have roles in maintaining BM homeostasis from fetal to adult development [130]. For example, HSCs form a unique homeostatic BM niche microenvironment and Nestin^+^ MSCs, identified using nestin expression, are an essential BM niche component [131], thus suggesting an interactive unit between the both stem cells to maintain bone/BM homeostasis. Generally, to replace lost bone due to defects in bone formation is required for the maintenance of stable bone mass throughout adulthood under physiological conditions. Normal bone homeostasis is maintained through a dynamic balance involved between osteoblast and osteoclast [132–134]. MSCs, as the precursors of osteoblasts, have the potential of osteogenesis and chondrogenesis and, consequently, such cells can contribute to bone remodeling. Osteoclasts are derived from the haematopoietic lineage [132, 134], likely to initially arise from the yolk sac [135]. Dysregulation of bone homeostasis has been linked to age-related bone loss, especially in postmenopausal women [133, 134, 136], potentially leading to osteoporosis. During skeletal development, bone formation appears to be in two distinct processes: intramembranous and endochondral ossification involved complex multiple signaling pathways [134, 135, 137]. Intramembranous ossification occurs via direct osteoblast differentiation in the absence of a cartilage structure while endochondral ossification is involved in the formation of cartilage tissue and subsequent replacement of this cartilage with mineralized bone [134, 135, 137, 138]. The vascular invasion by different mechanisms during intramembranous and endochondral ossification has been documented by multiple studies [139–141]. For example, an early in vivo study demonstrated that connective tissue growth factor (CTGF) is likely to regulate chondrocyte proliferation, extracellular matrix synthesis and angiogenesis [140]. This study by Ivkovic et al. [140] showed that an impairment of endochondral ossification associated with the decreased vascular endothelial growth factor in the ossification zone of the growth plates in *Ctgf*^*−/−*^ mice. Normally, bone/BM microenvironment is a physiological microenvironment and resident or recruited MSCs contribute to maintaining BM homeostasis. However, MSCs, in response to physiological chance or diseases, may exert diverse effects on surrounding dynamic BM microenvironment.

## Heterogeneity of MSCs and functional differences in various tissue-derived MSCs

### Heterogeneity of MSCs

Heterogeneity of MSCs, including donor-to-donor and cell–cell heterogeneity, is inherent and results from donor variation, such as donor age, gender, tissue source and health status, and the surrounding microenvironment, such as inflammation and disease status. The functions and characteristic of MSCs can be affected by the environmental factors. For example, a previous study demonstrated that interferon-γ induced the high expression of HLA class II in undifferentiated but not differentiated MSCs [142]. Varghese et al. [143] conducted a systematic review of patients’ factors (disease) affecting AT-derived MSCs’ viability as well as their functions. Proliferation and differentiation of MSCs were decreased with patient factors highlighted in this study [143] such as increasing age, body mass index, DM and exposure to radiotherapy and Tamoxifen. Wang et al. discussed with the possible effects of individual SNPs that are involved in monogenetic or multi-factorial diseases and the authors of this study did not suggest stem cell transplantation into the recipients with the same disease once stem cells carrying disease-associated SNPs [144]. Nowadays, new technologies and facilities are accessible for association analysis of disease-associated SNPs, such as stem cell tissue sources, genetic variants, gene modifications and next-generation sequencing [16, 144]. Therefore, clinical therapies using MSCs from self or donors with known or suspected disease susceptibility-related genetic background may not benefit recipients to treat diseases or conditions in the long term. Potential complications connected with the abnormality of MSCs’ biology may increase since transplanted stem cells may remain for many years in MSC transplant recipients.

Heterogeneity of MSCs often impacts their therapeutic potency and stable therapeutic outcomes and, therefore, it is essential to develop new ways to reduce the heterogeneity of MSCs. Given the heterogeneity of MSCs related to a heterogeneous cell mixture during isolation and culture-expanded preparation of MSCs [145, 146], one strategy to reduce heterogeneity is the utilization of single colony forming unit-derived colonies of MSCs to expand and obtain the final stem cell products [147]. Furthermore, it has been noted that the emergence of heterogeneity in MSC populations originating from single-cell-derived colonies [148]. The use of the subpopulations of MSCs may be an effective approach to maximize the homogeneity of MSC products. For example, vascular cell adhesion molecule (VCAM)-1^+/−^ MSCs isolated from placenta chorionic villi (CV) are separated by Flow Cytometry and the subpopulation of VCAM-1^+^ CV-MSCs display potent pro-angiogenic activity [149]. Still, cell isolation and culture conditions need to be precisely standardized for culture-expanded MSCs to control product consistency. Therefore, management of functional heterogeneity of MSCs across different donors and subpopulations of MSCs should be considered for more safe and efficacious MSC-based therapies in the clinical settings.

### Specific therapeutic effects of MSCs obtained from different sources

The MSC source becomes important in preclinical and clinical applications. Due to the diverse tissue-specific properties of MSCs along with the diverse MSC-associated special tissue microenvironments, MSCs obtained from different types of tissues have different functional behaviors. There are important differences in the characteristics of BM-derived MSCs and AT-derived MSCs reported in a previous study [150]. Importantly, BM-derived MSCs demonstrate the higher potential for differentiation into osteoblasts and lower adipogenesis potential compared to AT-derived MSCs [150]. In an in vivo study, UC-derived MSCs shows the strongest therapeutic efficacy among the three types of MSCs in regulation of fasting blood glucose in T2DM mice, followed by dental pulp-derived MSCs that display an intermediate efficacy as well as the least efficacy of therapy with AD-derived MSCs [151]. In addition, there are various expression of paracrine action in different sources of MSCs, although MSCs from different source have similar functional properties such as anti-inflammation. Previous studies have shown that extracellular vesicles (EVs) secreted from different MSC sources have specific functions and therapeutic effects [152, 153], thus reflecting paracrine differences between MSCs from different tissue sources. For example, EVs secreted by human BM-derived MSCs can promote cartilage regeneration and osteoarthritis in vitro [154, 155], suggesting that EVs from BM-derived MSCs have specific osteoinductive potential. Taken together, further work needs to identify an optimal source of MSCs as a priority for their safe and efficacious clinical applications, while acknowledging that MSCs from different sources have been seen different functional properties in vitro.

## Autologous vs. allogeneic MSCs: therapeutic limitation

**Fig. 2:**
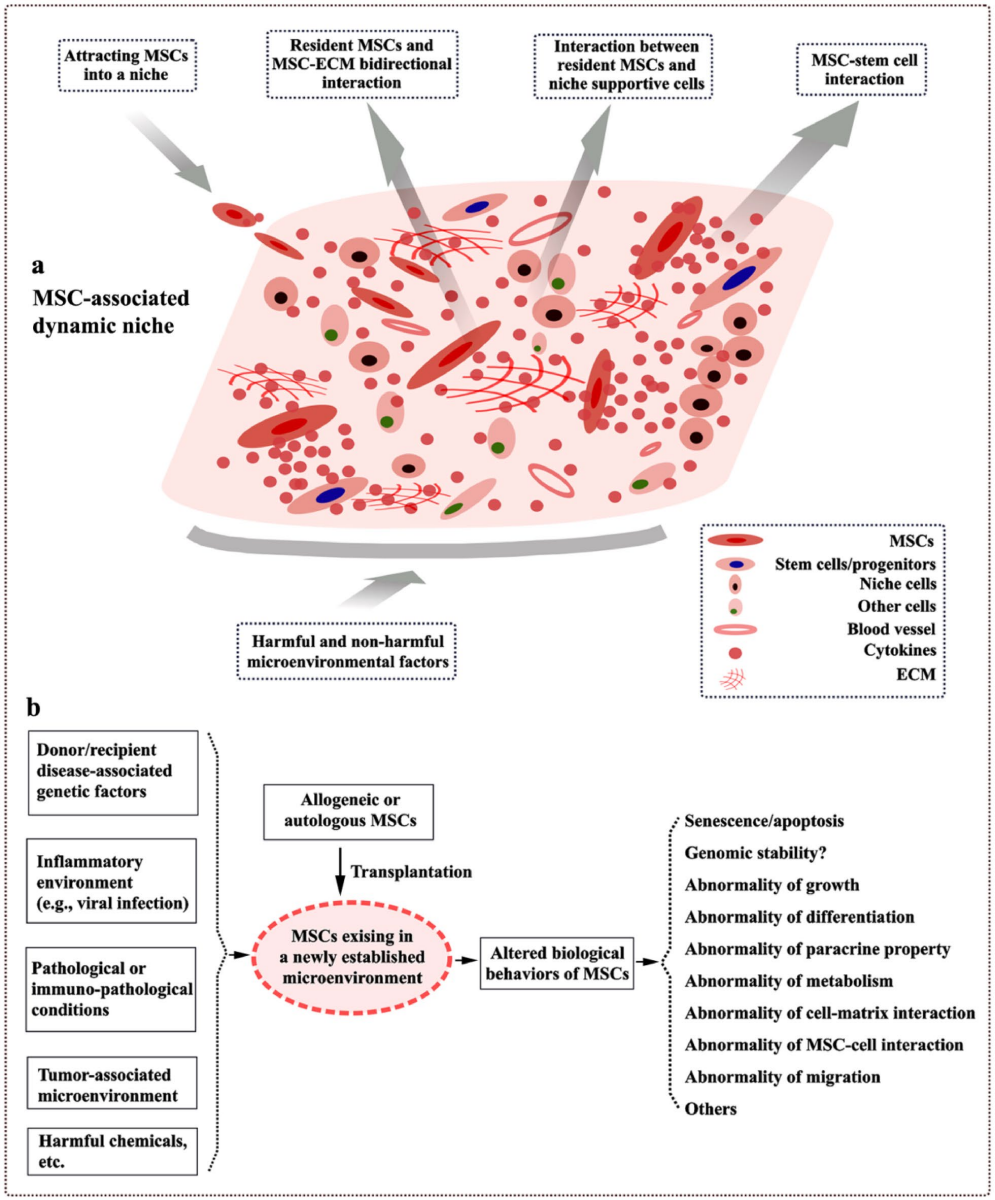
Bidirectional interaction between MSCs and MSC-surrounding dynamic microenvironment. **a** MSC-existing microenvironments in the recipients are composed of the diverse cellular subpopulations as well as the niche-associated stroma. Bidirectional interaction is noted between MSCs and MSCs’ microenvironment contents. **b** Allogeneic or autologous MSC transplant has been used for the treatment of diseases and conditions. Such MSCs in the transplanted tissue may be potentially impacted by the diverse pathological microenvironmental factors and, consequently, MSCs’ biological behaviors are probably altered in the recipients

**Fig. 3:**
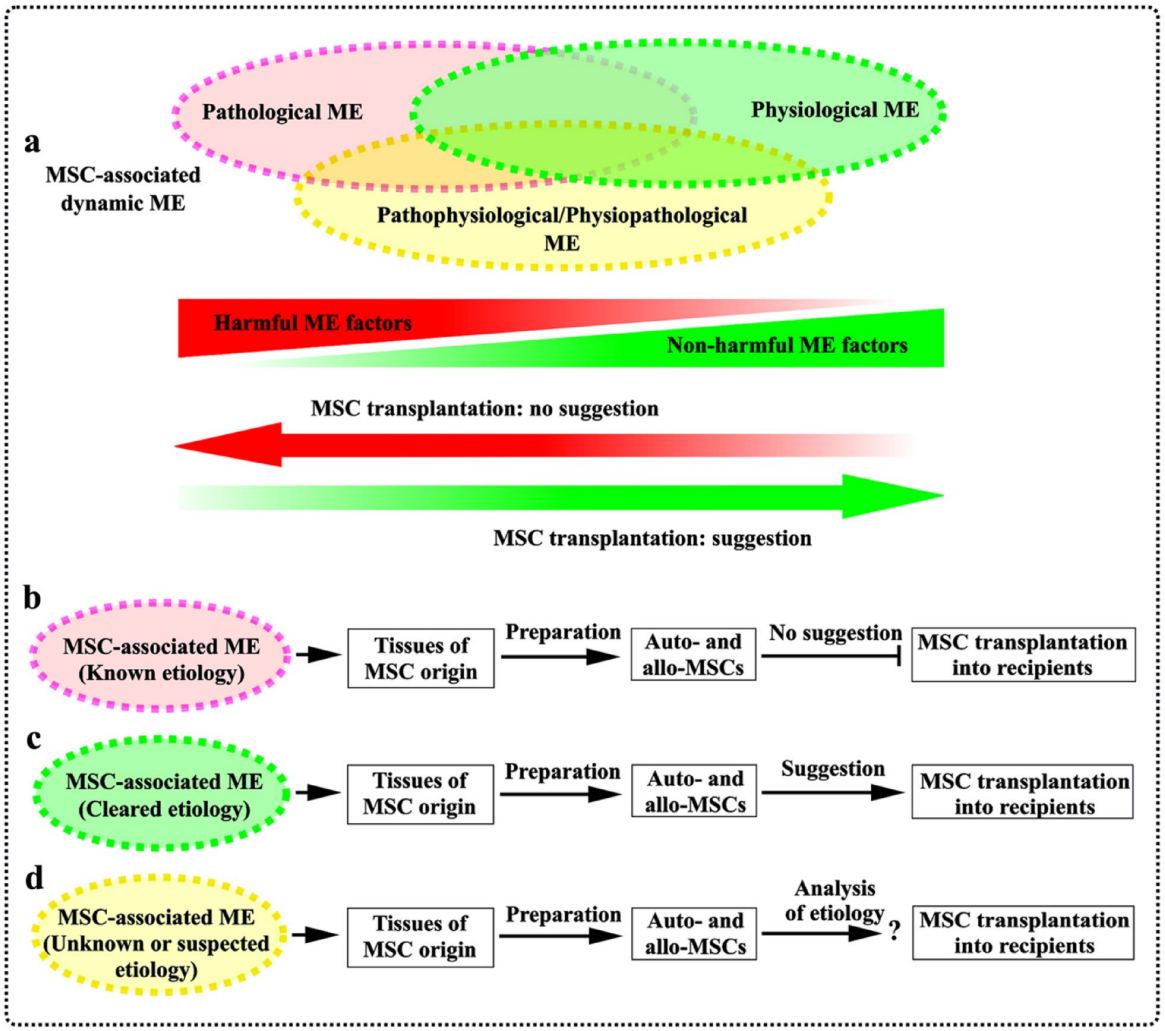
MSC-associated heterogeneous microenvironments and MSC therapeutic suggestion. **a** The various MSC-associated dynamic microenvironments have been sensed within the body, as illustrated by pathological, physiological and/or pathophysiological/physiopathological microenvironments. A newly established tissue microenvironment may be impacted and altered by pathological or physiological environmental factors. **b** Biological behaviors of MSCs resided in a special tissue may be affected by the pathological harmful microenvironmental factors. Such MSCs obtained from the special tissue are not suggested to be transplanted into the recipients. **c** MSCs obtained from healthy tissues can be transplanted into the recipients. **d** MSCs may also exist in an unknown or suspected etiological microenvironment. MSCs obtained from a special tissue, where MSCs are potentially impacted by unknown or suspected harmful microenvironmental factors, are suggested to be conducted an analysis of etiology for their medical practice. *Allo* allogeneic, *Auto* autologous, *ME* microenvironment

Autologous or allogeneic MSCs, in response to local microenvironment cues after infusion, are thought to possibly affect their functional properties. Therefore, safety and efficacy within different contexts need to be further considered in MSC-based therapies. From the present data available, it is possible to draw a figure demonstrating our current understanding of the bidirectional interaction between MSCs and MSCs’ microenvironmental contents (Fig. 2a). The potential impact of MSCs by MSC-surrounding microenvironment should be considered whether to support the potential use of patient-derived autologous MSCs, even the allogeneic, for disease treatment (Fig. 2b). Due to potential harmful or non-harmful microenvironmental factors, MSC-associated physical microenvironments are complex in tissues, which is supposed to be roughly categorized as the pathological, physiological, or pathophysiological (Fig. 3a). Potentially impacted tissue-derived MSCs populations by pathological microenvironments are not suggested for clinical applications (Fig. 3b). For example, juvenile idiopathic arthritis (JIA) is known as juvenile rheumatoid arthritis and specific genetic susceptibility genes have been identified, which are divided into the HLA genes and non HLA-related genes [156]. Instead of using autologous BM-derived MSCs, the use of allogeneic BM-derived MSCs [157] and UC-derived MSCs [158] has reported for a potentially safe and effective treatment option for JIA. Using autologous AT-derived MSCs may also be an effective therapeutic option for JIA. When MSCs are existing in or surrounding an unknown or suspected etiological microenvironment, association analysis of disease etiology (e.g., disease-associated SNPs) may be advisable in MSC transplantation for personalized therapies (Fig. 3d).

## Summary and conclusions

The plasticity and functional heterogeneity of MSCs may raise potential questions in MSC-based safe and efficacious therapies in the clinical applications. Acknowledging a connection between the biological properties of MSCs and MSC-associated microenvironmental factors is conducive for better understanding of MSCs’ contribution to their medical practice, promisingly or uncertainly. As of March 2021, there are almost 1000 clinical trials registered on the clinicaltrials.gov (www.clinicaltrials.gov) [159] using autologous and allogeneic MSCs for treatment of the variety of categories of human diseases and medical conditions. Clinical data available show that the therapeutic benefits of using either autologous or allogeneic MSCs as a better option are inconclusive. Clinical application using MSCs from self or donors has been long debated with a focus on genetic etiologies involved in monogenetic or multi-factorial diseases. MSCs from self or even donors with known or suspected disease susceptibility-related genetic background may not benefit recipients to treat diseases or conditions in the long term because such cells may remain in the recipient body for many years. Complications connected with MSCs’ abnormal biological behaviors may increase in recipients, which may impact on the long-term detrimental functional consequences within the body. On an individual therapeutic basis, donor-control clinical practice, in particular association analysis of disease-associated SNPs in MSCs, is suggested to further consider for the safe and effective therapies for the MSC transplant recipients.

## Data Availability

Not applicable.

## Abbreviations

AGIA: Gastrointestinal adenocarcinoma
ARDS: Acute respiratory distress syndrome
AT: Adipose tissue
BM: Bone marrow
BMC: Bone marrow cells
COVID-19: Coronavirus disease 2019
CTGF: Connective tissue growth factor
CV: Chorionic villi
DCM: Dilated cardiomyopathy
DM: Diabetes mellitus
EVs: Extracellular vesicles
GCV: Ganciclovir
GVHD: Graft-versus-host disease
HSCs: Hematopoietic stem cells
ICU: Intensive care unit
IPF: Idiopathic pulmonary fibrosis
ISCT: International Society for Cellular Therapy
JIA: Juvenile idiopathic arthritis
MI: Myocardial infarction
MHC: Major histocompatibility complex
MSC: Mesenchymal stem/stromal cell
OA: Osteroarthritis
PBPCs: Peripheral blood progenitor cells
RFMSCs: Retropatellar fat pad-derived MSCs
SLE: System lupus erythematosus
SLEDAI: SLE disease activity index
SNP: Single-nucleotide polymorphism
SSc: Systemic sclerosis
T1DM: Type 1 diabetes mellitus
T2DM: Type 2 diabetes mellitus
TME: Tumor microenvironment
UC: Umbilical cord
VCAM: Vascular cell adhesion molecule
WJ: Wharton’s jelly
